# Maternal zinc deficiency during pregnancy elevates the risks of fetal growth restriction: a population-based birth cohort study

**DOI:** 10.1038/srep11262

**Published:** 2015-06-08

**Authors:** Hua Wang, Yong-Fang Hu, Jia-Hu Hao, Yuan-Hua Chen, Pu-Yu Su, Ying Wang, Zhen Yu, Lin Fu, Yuan-Yuan Xu, Cheng Zhang, Fang-Biao Tao, De-Xiang Xu

**Affiliations:** 1 School of Public Health, Anhui Medical University, Hefei, China; 2 Anhui Provincial Key Laboratory of Population Health & Aristogenics, Anhui Medical University, Hefei, China

## Abstract

We investigated the association between maternal zinc level during pregnancy and the risks of low birth weight (LBW) and small for gestational age (SGA) infants in a large population-based birth cohort study. In this study, 3187 pregnant women were recruited. For serum zinc level, 2940 pregnant women were sufficient (≥56 μg/dL) and 247 deficient (<56 μg/dL). Of interest, 7.3% newborns were with LBW among subjects with low zinc level (*RR*: 3.48; 95% *CI*: 2.03, 5.96; *P* < 0.001). Adjusted *RR* for LBW was 3.41 (95% *CI*: 1.97, 5.91; *P* < 0.001) among subjects with low zinc level. Moreover, 15.0% newborns were with SGA among subjects with low zinc level (*RR*: 1.98; 95% *CI*: 1.36, 2.88; *P* < 0.001). Adjusted *RR* for SGA was 1.93 (95% *CI*: 1.32, 2.82; *P* < 0.001) among subjects with low zinc level. A nested case-control study within above cohort showed that maternal serum zinc level was lower in SGA cases as compared with controls. By contrast, maternal serum C-reactive protein, TNF-α and IL-8 levels were significantly higher in SGA cases than that of controls. Moreover, nuclear NF-κB p65 was significantly up-regulated in placentas of SGA cases as compared with controls. Taken together, maternal zinc deficiency during pregnancy elevates the risks of LBW and SGA infants.

Fetal growth restriction (FGR), which manifests as low birth weight (LBW) or small for gestational age (SGA), increases infant mortality and morbidity^1^,^2^. Almost 25 years ago, Barker and coworkers described FGR as highly correlated with increased risk for the development of cardiovascular diseases during adulthood^3^,^4^. Since then, numerous epidemiologic studies have demonstrated an association between FGR and an increased risk for adult onset of metabolic as well as non-metabolic diseases^5^,^6^. Thus, the etiology and underlying mechanism for FGR is of great concern.

Zinc (Zn) is a structural constituent that is essential for cell growth, development and differentiation^7^. Several earlier reports demonstrate that maternal zinc deficiency during pregnancy is linked with adverse pregnant outcomes including abortion, preterm delivery, stillbirth and fetal neural tube defects^8^,^9^,^10^,^11^. A double-blind and randomized controlled study shows that zinc supplementation during pregnancy improves birth length after adjusting for maternal height, pre-pregnancy weight and parity^12^. According to a recent systematic review, prenatal zinc supplementation leads to a statistically significant lower incidence of preterm birth^13^,^14^,^15^. The link between maternal zinc status during pregnancy and birth weight has been investigated in several small epidemiological studies^16^,^17^. Nevertheless, the association between maternal zinc status during pregnancy and the incidences of LBW and SGA infants needs to be determined in a large longitudinal investigation.

The objective of the present study was to assess maternal serum zinc level during pregnancy in a large population-based birth cohort study. We were to further analyze the association between maternal serum zinc level at different gestational stages and the risks of LBW and SGA infants. We also sought to explore the link among maternal serum zinc level during pregnancy, placental inflammation and the incidence of LBW and SGA infants.

## Results

**Figure 1:**
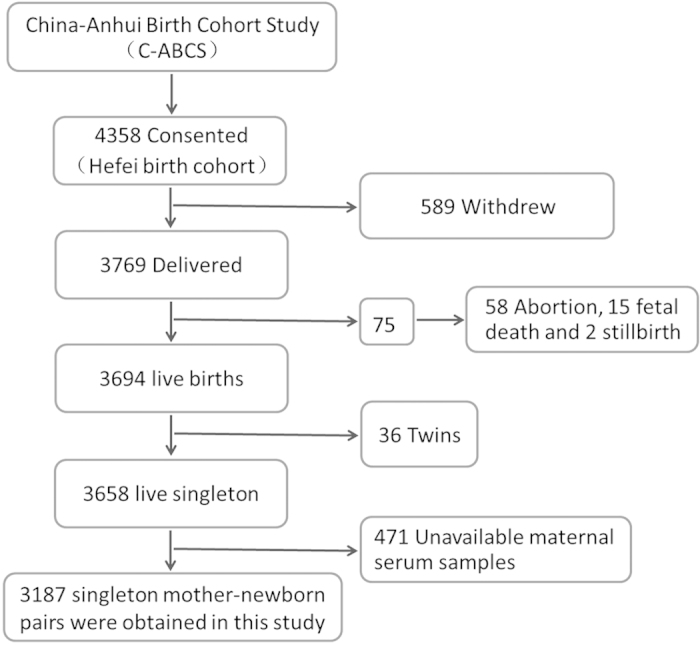
Flow diagram of recruitment and follow-up in this birth cohort study.

In the present study, 3187 pregnant women were recruited (Fig. 1). The average serum zinc concentration was 91.0 μg/dL among 3187 pregnant women. For serum zinc level, 2940 pregnant women were sufficient (≥56 μg/dL) and 247 deficient (<56 μg/dL). No subject was drinking alcohol or smoking cigarette during pregnancy (data not shown). The demographic characteristics of pregnant women and their newborns were compared between subjects with normal zinc level and low zinc level. No significant difference on mother’s age, BMI before pregnancy and monthly income per person was observed between two groups (Table 1). As expected, 96.2% of subjects with normal zinc level and 92.3% of subjects with low zinc level were primiparous. The average gestational age at birth was 39.1 weeks among subjects with normal zinc level and 39.1 weeks among subjects with low zinc level, respectively. There was no significant difference on gestational age at birth between two groups (Table 1). The average birth weight was 3401 g among subjects with normal zinc level and 3357 g among subjects with low zinc level, respectively. There was a downward trend on the average birth weight in subjects with low zinc level as compared with that of subjects with normal zinc level (*P* = 0.210). Further analysis showed that several maternal characteristics, including maternal age, monthly income, parity and gravidity, did not influence serum zinc level during pregnancy (Table 2). Of interest, the average serum zinc levels were significantly lower among subjects with BMI < 18.5 kg/m^2^ than those of subjects with normal BMI (18.5–24.9 kg/m^2^) (Table 2).

The association between serum zinc level during pregnancy and the risk of LBW infants was analyzed. As shown in Table 3, 2.2% newborns were with LBW among subjects with normal zinc level. Moreover, 7.3% newborns were with LBW among subjects with low zinc level (*RR*: 3.48; 95% *CI*: 2.03, 5.96; *P* < 0.001). Adjusted *RR* for LBW was 3.41 (95% *CI*: 1.97, 5.91; *P* < 0.001) among subjects with low zinc level using multiple logistic regression model (Table 3). The association between maternal zinc level during early gestational stage and the risk of LBW infants was further analyzed. As shown in Table 4, 2.6% newborns were with LBW among subjects with normal zinc level during the first trimester. Of interest, 6.5% newborns were with LBW among subjects with low zinc level during the first trimester (*RR*: 2.63; 95% *CI*: 0.58, 12.00; *P* = 0.211). Adjusted *RR* for LBW was 2.64 (95% *CI*: 0.58, 12.05; *P* = 0.211) among subjects with low zinc level during the first trimester (Table 4). The association between maternal zinc level during later gestational stages and the risk of LBW infants was analyzed. As shown in Table 5, 2.1% newborns were with LBW among subjects with normal zinc level during the second and third trimesters. Of interest, 7.4% newborns were with LBW among subjects with low zinc level during the second and third trimesters (*RR*: 3.70; 95% *CI*: 2.06, 6.62; *P* < 0.001). Adjusted *RR* for LBW was 3.81 (95% *CI*: 2.12, 6.85; *P* < 0.001) among subjects with low zinc level during the second and third trimesters (Table 5).

The association between zinc level during pregnancy and the risk of SGA infants was then analyzed. As shown in Table 3, 8.2% newborns were with SGA among subjects with normal zinc level. Of interest, 15.0% newborns were with SGA among subjects with low zinc level (*RR*: 1.98; 95% *CI*: 1.36, 2.88; *P* < 0.001). Adjusted *RR* for SGA was 1.93 (95% *CI*: 1.32, 2.82; *P* < 0.001) among subjects with low zinc level (Table 3). The association between zinc level during early gestational stage and the risk of SGA infants was then analyzed. As shown in Table 4, 8.3% newborns were with SGA among subjects with normal zinc level during the first trimester. Of interest, 22.6% newborns were with SGA among subjects with low zinc level during the first trimester (*RR*: 3.23; 95% *CI*: 1.33, 7.84; *P* = 0.009). Adjusted *RR* for SGA was 3.12 (95% *CI*: 1.27, 7.66; *P* = 0.013) among subjects with low zinc level during the first trimester (Table 4). The association between zinc level during later gestational stages and the risk of SGA infants was then analyzed. As shown in Table 5, 8.1% newborns were with SGA among subjects with normal zinc level during the second and third trimesters. Of interest, 13.9% newborns were with SGA among subjects with low zinc level during the second and third trimesters (*RR*: 1.82; 95% *CI*: 1.21, 2.76; *P* = 0.004). Adjusted *RR* for SGA was 1.82 (95% *CI*: 1.20, 2.75; *P* = 0.005) among subjects with low zinc level during the second and third trimesters (Table 5).

**Figure 2:**
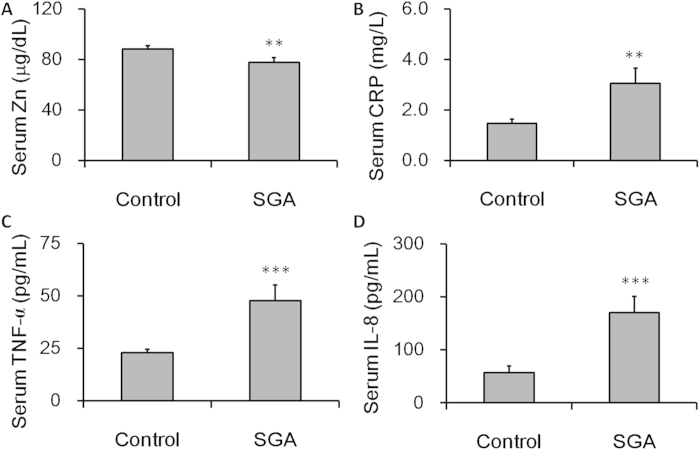
Serum zinc level during early gestational stage and inflammatory cytokines in maternal sera from SGA cases and controls. Serum zinc level and inflammatory cytokines during the first trimester were measured in 50 SGA cases and 100 controls. (**A**) Zinc. (**B**) CRP. (**C**) TNF-α. (**D**) IL-8. All data were expressed as *means ± SEM*. **P < 0.01, ***P < 0.001 as compared with controls.

We compared serum zinc level from SGA cases and controls. As expected, the level of serum zinc during pregnancy was lower in SGA cases as compared with controls (Fig. 2A). We then compared the levels of CRP, TNF-α and IL-8 in maternal sera from SGA cases and controls. As shown in Fig. 2B, the level of CRP in maternal sera was significantly higher in SGA cases than that of controls. In addition, the level of TNF-α and IL-8 in maternal sera was also higher in SGA cases than that of controls (Fig. 2C,D).

**Figure 3:**
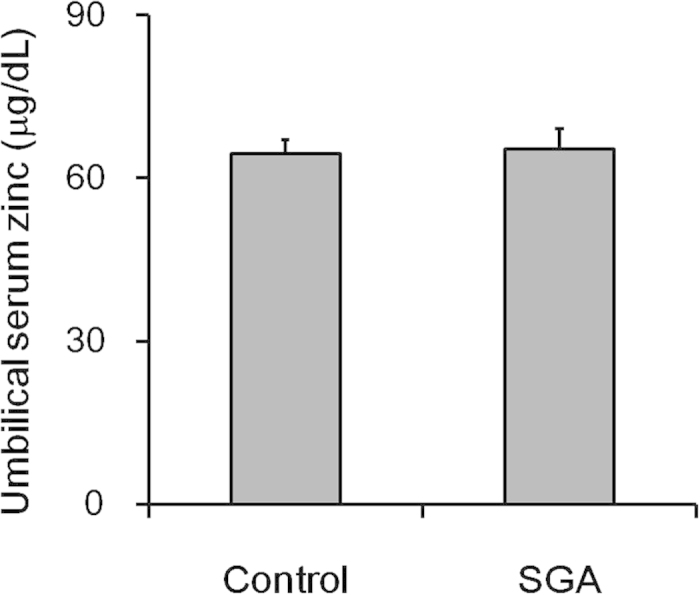
Zinc level in umbilical sera from SGA cases and controls. Zinc level in umbilical sera was measured in 30 SGA cases and 30 controls. All data were expressed as *means ± SEM*.

**Figure 4:**
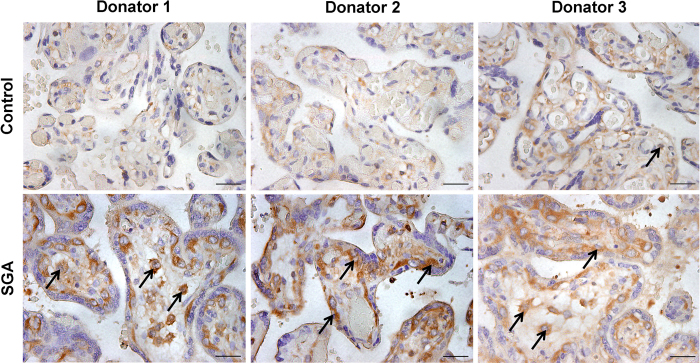
NF-κB p65 in placentas from SGA cases and controls. Placental NF-κB p65 was measured in controls and SGA cases using immunohistochemistry. Arrows indicate NF-кB p65-positive cells. Scale bar: 50 μm.

We compared zinc level in umbilical sera from 30 SGA cases and 30 controls. Unexpectedly, there was no significant difference on the level of zinc in umbilical sera between SGA cases and controls (Fig. 3). We then compared the expression of placental NF-κB p65 between SGA cases and controls. As expected, nuclear NF-κB p65 was significantly up-regulated in placentas of SGA cases as compared with controls. Furthermore, nuclear translocation of NF-kB p65 was mainly observed in trophoblasts of SGA placenta (Fig. 4).

## Discussions

The present study assessed serum zinc concentration among 3187 pregnant women. Our results showed that the average serum zinc concentration during pregnancy for all subjects was 91.0 μg/dL. According to the suggested lower cutoffs of serum zinc concentrations by NHANES II^18^, 2940 pregnant women (92.2%) were sufficient (≥56 μg/dL) and 247 (7.8%) deficient ( <56 μg/dL). These results are similar to the results from NHANES in 1976–1980^18^. Several reports demonstrate that socio-demographic status and economic income are associated with zinc deficiency^19^,^20^. In addition, age and gender are important determinants of serum zinc concentrations^21^. The present study investigated the effects of maternal age, monthly income, parity and gravidity on serum zinc concentration during pregnancy. Surprisingly, these factors had little effect on serum zinc concentration during pregnancy. According to several earlier reports, maternal serum or plasm zinc concentrations declined as pregnancy progressed^18^,^22^,^23^. Of interest, the present study showed that the average zinc levels were significantly lower among subjects with BMI < 18.5 than those of subjects with BMI18.5–24.9. The inconsistency of the present results with past findings may be related to following reasons: first, different suggested lower cutoffs of serum zinc concentrations were chosen; sencond, pregnant woman population with different age, BMI, average monthly income, and gestational ages was investigated; third, past results came most frequently from small samples. To our knowledge, the present study is the first to assess serum zinc concentration during pregnancy in a large population-based birth cohort study.

It remains contradictory whether maternal zinc deficiency during pregnancy elevates the risks of LBW and SGA infants. An earlier study demonstrated that there was a threshold below which the prevalence of LBW infants was increased^17^. By contrast, two recent reports found that maternal zinc level during pregnancy was not significantly associated with the incidence of LBW and SGA infants^24^,^25^. In the present study, we showed that maternal zinc deficiency during pregnancy elevated the risks of LBW and SGA births in a large population-based birth cohort study. This result will provide a scientific basis for zinc to prevent or control LBW and SGA especially in high-risk pregnant women with zinc deficiency. The next step is to determine whether maternal zinc supplementation during pregnancy reduces the risks of LBW and SGA infants.

Until now, no report analyzed the effects of zinc deficiency at different gestational stages on fetal growth. The present study compared the effects of zinc deficiency at different gestational stages on the incidence of LBW and SGA infants. Results suggest that maternal zinc deficiency at different gestational stages produces differential effects on fetal growth. Maternal zinc deficiency during early gestational stage elevates the risk of SGA infants, while zinc deficiency during later gestational stages results in the incidence of LBW and SGA infants. In addition, no significant difference in the level of zinc in umbilical sera is observed between SGA cases and controls. Although the confirmation for this result requires a larger sample size, the current result suggests that FGR, caused by maternal zinc deficiency during pregnancy, may not be attributed to zinc deficiency in the fetus.

Increasing evidence has demonstrated that zinc has an anti-inflammatory effect^26^. An earlier study found that injection with zinc during pregnancy ameliorated inflammation-associated teratogenicity in mice^27^. According to a recent report from our laboratory, oral supplementation with zinc protected mice from LPS-induced IUGR through its anti-inflammatory activity^28^. The present study showed that maternal serum zinc concentration was significantly lower in SGA cases than that of controls. In contrast with low zinc level, maternal serum CRP, TNF-α and IL-8, three important inflammatory cytokines, were markedly higher in SGA cases than those of controls. Correspondingly, NF-κB, a regulator of inflammatory cytokines, was activated in placentas from SGA cases. Thus, it is reasonable to speculate that activation of placental NF-κB signaling and elevation of serum inflammatory cytokines are attributed to low zinc level in SGA infants. Indeed, several recent studies showed that inflammatory cytokines were elevated in the cord blood from SGA infants^29^,^30^. Taken together, these results suggest that there is an association between placental inflammation and the incidence of SGA infants among subjects with low zinc level during pregnancy.

In the present study, we laid emphasis on the association of maternal zinc deficiency during pregnancy and the risks of LBW and SGA infants. The present study has some limitations. First, the present study only analyzed the effects of maternal zinc deficiency during pregnancy on fetal growth. Indeed, several reports demonstrated that maternal deficiency of other micronutrients, such as folate and vitamin B-12, elevated the risks of LBW and SGA infants^31^,^32^. Supplementation with either folic acid or multivitamins reduced the risk of SGA infant^33^,^34^. Second, the present study did not clarify the mechanism why maternal zinc deficiency during pregnancy elevated the risks of LBW and SGA infants. Additional work is required to determine whether maternal zinc supplementation during pregnancy reduces the risks of LBW and SGA infants and other adverse pregnancy outcomes. In addition, the mechanism by which maternal zinc deficiency during pregnancy induces FGR needs to be explored in animal experiments.

In summary, the present study investigated the association between maternal zinc level during pregnancy and the risks of LBW and SGA infants in a large population-based birth cohort study. The present results allow us to reach the following conclusions. First, maternal zinc deficiency during pregnancy elevates the risks of LBW and SGA infants; second, maternal zinc deficiency during early gestational stage elevates the risk of SGA infant, while zinc deficiency during later gestational stages results in the incidence of LBW infant; third, there is an association among maternal serum zinc level, placental inflammation and the incidence of SGA infants.

## Methods

### Study design

To avoid repeated freeze-thaw cycles for all serum samples and to ensure sufficient sample sizes, the present study analyzed a subsample of the China-Anhui Birth Cohort Study (C-ABCS) cohort^35^ that recruited 4358 pregnant women from Hefei city of Anhui province from January 1 to December 31 in 2009. For this study, eligible participants were mother-and-singleton -offspring pairs in which serum samples from mothers were available for analysis of serum zinc level and offspring had a detailed birth records. Thirty-six pregnant women giving birth to twins, 15 fetal deaths, 2 stillbirths, 58 abortions and 589 withdrew were excluded from this study. In addition, 471 cases were also excluded from this study due to maternal sera exhausted for other experiments (Fig. 1). Total 3187 mother-and-singleton-offspring pairs were eligible for this study. All neonates were weighed at birth. The present study was approved by the ethics committee of Anhui Medical University. The methods were carried out in accordance with the approved guidelines. Oral and written consents were obtained from all pregnant women.

### Definition of SGA and LBW

In this study, SGA births were live-born infants that were <10^th^ percentile of birth weight according to nomograms based on gender and gestational age from a reference population of 13,454 infants delivered at C-ABCS^36^. LBW births were live-born infants that were less than 2500 g for birth weight.

### Nested case-control study

To analyze the association among serum zinc status, inflammatory cytokines and FGR, a nested case-control study within above cohort were designed. In the case-control study, 50 SGA cases and 100 controls were randomly chosen. For control subjects, a common control series (case: control = 1:2) was selected from the non–case subjects in this cohort. The controls were matched with these cases with regard to pre-pregnancy BMI, maternal age, time for collecting serum and average monthly income. The present study obtained ethics approval from the ethics committee of Anhui Medical University. Oral and written consents were obtained from all pregnant women.

### Collection of placentas and umbilical sera

To compare the expression of placental NF-κB p65 in SGA cases and controls, a small case-control study was designed. Thirty SGA placentas and 30 control placentas were collected by the Maternal and Child Care Service Centre of Maanshan city (Maanshan, Anhui province, China). To analyze the association between zinc level in umbilical sera and IUGR, umbilical sera were also collected from 30 SGA cases and 30 controls by the Maternal and Child Care Service Centre of Maanshan city. The controls were matched with these cases with regard to pre-pregnancy BMI, maternal age, socio-economic status, parity and gestational week of collecting serum. The present study obtained ethics approval from the ethics committee of Anhui Medical University. Oral and written consents were obtained from all pregnant women.

### Measurement of serum zinc

Maternal fasting blood during pregnancy was collected in the morning. The blood samples kept under refrigeration were allowed to clot for 30 mins. Then maternal serum was obtained after centrifuging for 15 mins at 3000 g. After discarding hemolytic specimens, available sera were stored at –80 ºC until analysis. To avoid contaminaion of exogenous zinc, all centrifuge tubes, storage vials and transfer pipettes were soaked for 24 hrs in ultrapure 10% HNO_3_ at room temperature. Serum zinc concentration was determined by flame atomic absorption spectroscopy (FAAS) as previously described^37^. Serum samples were diluted with 1% HNO_3_ according to 1:35 (v/v). The diluted solution was then detected using FAAS. Each sample was analyzed in triplicate. Precision of the method was measured by coefficients of variation. Mean CV for measurement of serum zinc was 4.9% for within-day determinations and 4.3% for day-to-day determinations. The detection limit of this method was 0.2 μg/dL.

### Enzyme-linked immunosorbent assay (ELISA)

Commercial human TNF-α and IL-8 ELISA kits (R&D Systems, Abingdon, Oxon, UK) and human C-reactive protein (CRP) ELISA kits (TSZ ELISA, Waltham, MA, USA) were used to measure TNF-α, IL-8 and CRP in maternal serum according to the manufacturer’s protocols.

### Immunohistochemistry

Human placenta tissues from SGA cases and controls were fixed in 4% paraformaldehyde and embedded in paraffin according to the standard procedure. Paraffin embedded tissues were cut 5 μm thick and stained with hematoxylin and eosin (H & E) for morphological analysis. For immunohistochemistry, paraffin-embedded placental sections were deparaffinized and rehydrated in a graded ethanol series. After antigen retrieval and quenching of endogenous peroxidase, sections were overnight incubated with NF-кB p65 monoclonal antibodies (1:200 dilution) at 4 °C. The color reaction was developed with HRP-linked polymer detection system and counterstaining with hematoxylin.

### Statistical analysis

Maternal serum zinc level during pregnancy was divided into two groups according to suggested criteria^18^: serum zinc concentration less than 56 μg/dL for zinc deficiency and serum zinc concentration more than 56 μg/dL for zinc sufficiency. Incidence and relative risk (*RR*) for LBW and SGA were calculated between two groups. For adjustment of pre-pregnancy BMI, maternal age, time for collecting serum and average monthly income, logistic regression model was used to estimate RR with 95% confidence intervals (95% *CI*) with respect to LBW and SGA incidence. All quantified data were expressed as means ± *SEM*. All statistical tests were two-sided using an alpha level of 0.05. ANOVA and the Student-Newmann-Keuls post hoc test were used to determine differences among different groups. Student *t* test was used to determine differences between two groups.

## Additional Information

**How to cite this article**: Wang, H. *et al.* Maternal zinc deficiency during pregnancy elevates the risks of fetal growth restriction: a population-based birth cohort study. *Sci. Rep.* **5**, 11262; doi: 10.1038/srep11262 (2015).

## Figures and Tables

**Table 1:** **Characteristics of 3187 mothers and their newborns**.

**Parameters**	**Maternal serum zinc level**^a^		***P***
	**Deficiency (n = 247)**	**Sufficiency (n = 2940)**	
*Maternal characteristics*			
Age (*y*, *means ± SD*)	27.1 ± 3.3	27.5 ± 3.2	0.092
≤24 [n(*%*)]	53 (21.5)	454 (15.4)	
25–29 [n(*%*)]	150 (60.7)	1850 (62.9)	0.032
≥30 [n(*%*)]	44 (17.8)	636 (21.6)	
BMI^b^ (*kg/m*^*2*^, *means ± SD*)	20.2 ± 2.3	20.2 ± 2.2	0.995
<18.5 kg/m^2^ [n(*%*)]	59 (23.9)	623 (21.2)	
18.5–24.9 kg/m^2^ [n(*%*)]	171 (69.2)	2143 (72.9)	0.461
>25 kg/m^2^ [n(*%*)]	17 (6.9)	174 (5.9)	
Parity [n(*%*)]			
1	228 (92.3)	2828 (96.2)	0.003
≥2	19 (7.7)	112 (3.8)	
Monthly income [n(*%*)]			
Low income^c^	119 (48.2)	1345 (45.7)	0.749
Middle income^c^	123 (49.8)	1538 (52.3)	
High income^c^	5 (2.0)	57 (1.9)	
*Newborn characteristics*			
Gestational age (*wk*, *means ± SD*)	39.1 ± 1.2	39.1 ± 1.4	0.954
Birth weight (*g*, *means ± SD*)	3357 ± 539	3401 ± 458	0.210
Time for collecting serum [n(*%*)]			
First trimester	48 (19.4)	1027 (34.9)	0.000
Second trimester	186 (75.3)	1833 (62.3)	
Third trimester	13 (5.3)	80 (2.7)	

^a^Deficiency for serum zinc<56 μg/dL, and sufficiency for serum zinc ≥ 56 μg/dL.

^b^BMI before pregnancy.

^c^Low income (1) for <2000 RMB per month; middle income (2) for ≥2000 RMB per month; high income (3) for ≥4000 RMB per month.

**Table 2:** **Influence of maternal characteristics on serum zinc level during pregnancy**.

**Characteristics**	***n***	**Maternal serum zinc (μg/dL,** ***means***** ± *****SD***)	***P***
Maternal age (*y*)			
≤24	507	92.8 ± 37.8	0.141
25–29	2000	90.2 ± 27.3	
≥30	680	92.0 ± 31.4	
Monthly income^a^			
Low	1464	90.5 ± 29.0	0.431
Middle	1661	91.3 ± 31.0	
High	62	94.9 ± 29.6	
BMI^b^ (*kg/m*^*2*^)			
<18.5	682	88.2 ± 28.6*	0.019
18.5–24.9	2314	91.7 ± 30.0	
≥25	191	92.8 ± 35.7	
Parity			
1	3056	91.1 ± 29.9	0.251
≥2	131	88.0 ± 35.4	
Gravidity			
1	1661	91.1 ± 30.1	0.768
≥2	1526	90.8 ± 30.1	

^a^Low income for <2000 RMB per month; middle income for ≥2000 RMB per month; high income for ≥4000 RMB per month.

^b^Pre-pregnancy BMI.

^*^P < 0.01 as compared with normal BMI (18.5–24.9 kg/m^2^).

**Table 3:** **The incidence and relative risk (** ***RR*** **) for LBW and SGA infants based on maternal serum Zn level**.

	**Maternal serum Zn level**^a^		***P***
	Deficiency (n = 247)	Sufficiency (n = 2940)	
LBW			
Number of LBW	18	65	
Incidence (*%*)	7.3	2.2	<0.001
Univariate *RR* (95%*CI*)	3.48 (2.03, 5.96)	1.00	<0.001
Adjusted *RR* (95%*CI*)^b^	3.41 (1.97, 5.91)	1.00	<0.001
SGA			
Number of SGA	37	240	
Incidence (*%*)	15.0	8.2	<0.001
Univariate *RR* (95%*CI*)	1.98 (1.36, 2.88)	1.00	<0.001
Adjusted *RR* (95%*CI*)^b^	1.93 (1.32, 2.82)	1.00	<0.001

^a^Deficiency for serum zinc < 56 μg/dL, and sufficiency for serum zinc ≥ 56 μg/dL.

^b^Adjusted for pre-pregnancy BMI, maternal age, gestational week for collecting serum and monthly income per person.

**Table 4:** **The incidence and relative risk (** ***RR*** **) for LBW and SGA infants based on maternal serum Zn level in the first trimester**.

	**Maternal serum Zn level**^a^		***P***
	Deficiency (n = 31)	Sufficiency (n = 627)	
LBW			
Number of LBW	2	16	
Incidence (*%*)	6.5	2.6	0.462
Univariate *RR* (95%*CI*)	2.63 (0.58, 12.00)	1.00	0.211
Adjusted *RR* (95%*CI*)^b^	2.64 (0.58, 12.05)	1.00	0.211
SGA			
Number of SGA	7	52	
Incidence (*%*)	22.6	8.3	0.007
Univariate *RR* (95%*CI*)	3.23 (1.33, 7.84)	1.00	0.009
Adjusted *RR* (95%*CI*)^b^	3.12 (1.27, 7.66)	1.00	0.013

^a^Deficiency for serum zinc < 56 μg/dL, and sufficiency for serum zinc ≥ 56 μg/dL.

^b^Adjusted for pre-pregnancy BMI, maternal age, and monthly income per person.

**Table 5:** **The incidence and relative risk (** ***RR*** **) for LBW and SGA infants based on maternal serum Zn level in the second and third trimesters**.

	**Maternal serum Zn level**^a^		***P***
	Deficiency (n = 216)	Sufficiency (n = 2313)	
LBW			
Number of LBW	16	49	
Incidence (*%*)	7.4	2.1	<0.001
Univariate *RR* (95%*CI*)	3.70 (2.06, 6.62)	1.00	<0.001
Adjusted *RR* (95%*CI*)^b^	3.81 (2.12, 6.85)	1.00	<0.001
SGA			
Number of SGA	30	188	
Incidence (*%*)	13.9	8.1	0.004
Univariate *RR* (95%*CI*)	1.82 (1.21, 2.76)	1.00	0.004
Adjusted *RR* (95%*CI*)^b^	1.82 (1.20, 2.75)	1.00	0.005

^a^Deficiency for serum zinc < 56 μg/dL, and sufficiency for serum zinc ≥ 56 μg/dL.

^b^Adjusted for pre-pregnancy BMI, maternal age, and monthly income per person.
